# Mast Cells and Natural Killer Cells—A Potentially Critical Interaction

**DOI:** 10.3390/v11060514

**Published:** 2019-06-04

**Authors:** Liliana Portales-Cervantes, Bassel Dawod, Jean S. Marshall

**Affiliations:** 1 Dalhousie Human Immunology and Inflammation Group, Dalhousie University, Halifax, NS B3H 4R2, Canada; lportales@dal.ca; 2 Department of Microbiology and Immunology, Dalhousie University, Halifax, NS B3H 4R2, Canada; 3 Department of Pathology, Dalhousie University, Halifax, NS B3H 4R2, Canada; dawod@dal.ca

**Keywords:** mast cells, NK cells, cytokines, CXCL8, interferons, cell recruitment, cellular activation, oncolytic virus

## Abstract

Natural killer (NK) cells play critical roles in host defense against infectious agents or neoplastic cells. NK cells provide a rapid innate immune response including the killing of target cells without the need for priming. However, activated NK cells can show improved effector functions. Mast cells are also critical for early host defense against a variety of pathogens and are predominately located at mucosal surfaces and close to blood vessels. Our group has recently shown that virus-infected mast cells selectively recruit NK cells and positively modulate their functions through mechanisms dependent on soluble mediators, such as interferons. Here, we review the possible consequences of this interaction in both host defense and pathologies involving NK cell and mast cell activation.

## 1. Mast Cells

Mast cells are highly specialized innate immune cells found in nearly all human tissues and in close proximity to blood vessels and nerves. Mast cells are able to respond to a variety of stimuli such as IgE-mediated activation [1], cytokines [2,3,4], neuropeptides [5], damage-associated molecular patterns [6], and hypoxic conditions [7]. Following activation, mast cells rapidly release an arsenal of pro-inflammatory mediators such as histamine, tryptase, and TNF, while synthesizing lipid mediators (e.g., leukotrienes and prostaglandins). In the longer term, mast cells produce a wide variety of cytokines and chemokines such as IL-13, IL-33, and CXCL8. Mast cell activities are often associated with the pathology of allergic diseases [8,9]. However, they have also been shown to be protective in host defense against a variety of venoms and to limit sterile inflammation [10,11,12,13]. Mast cells are also amongst the first immune cells to interact with invading pathogens due to their extensive presence at sites exposed to the external environment, such as the skin and mucosal surfaces. The direct contribution of mast cells to the clearance of bacteria and parasites has been demonstrated by multiple authors in experiments involving mast cell-deficient murine models [14,15,16,17,18]. Additionally, mast cells can modulate adaptive immune responses primarily through the mobilization and maturation of dendritic cells (DCs) but also through the impact of mast cell mediators directly and indirectly on T and B cell functions [19,20,21,22]. The contribution of mast cells to the host antiviral responses remains less well defined. Both beneficial and detrimental roles have been reported [23,24,25,26]. The latter has been associated with excessive inflammation and vascular leakage as a result of degranulation and/or expression of pro-inflammatory cytokines, while beneficial actions of mast cells have been associated with the recruitment of immune effector cells to sites of infection and their subsequent activation [27].

## 2. NK Cell–Mast Cell Interactions

The recruitment of NK cells to sites of infection is a complex process involving the upregulation of adhesion molecules and expression of chemotactic stimuli. Human mast cells infected with reovirus type 3 Dearing, which is frequently used in models of oncolytic virus therapy, can selectively recruit human NK cells by mechanisms largely dependent on chemokine production [28]. Mast cell–NK cell interactions can have a beneficial impact not only on the host antiviral responses but also in pathologies where NK cell actions are inhibited such as the solid tumor microenvironment. Often, NK cells are described to lyse target cells without any priming. However, cytokine-activated NK cells show enhanced cytotoxic activities, resulting in the recognition and killing of target cells that were not lysed by resting NK cells [29,30]. Therefore, stimuli provided by surrounding immune cells such as DCs [31,32] and mast cells [27,28,33] are critical for the most effective NK cell target identification and function. Cytokines recognized as positive modulators of NK cells functions include interferons (IFNs), IL-12, IL-15, IL-18, and IL-21 [34].

### 2.1. Viral Infection

**Figure 1:**
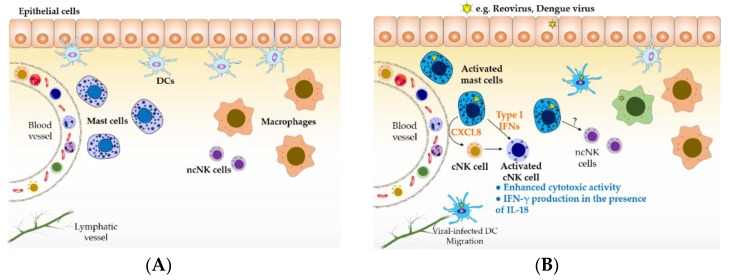
Mast cells sentinels of the immune system. (**A**) Tissue-resident mature mast cells are located at surfaces exposed to the external environment, where they can recognize invading pathogens, and in close proximity to blood vessels, where they can modulate the trafficking of immune cells into tissue. (**B**) During viral infections, multiple cells can become infected, resulting in the production of cytokines and chemokines involved in antiviral responses. Virus-infected mast cells can recruit conventional natural killer (NK) cells and induce their activation through the production of CXCL8 and type I interferons (IFNs), respectively. Type I IFN-activated NK cells better recognize target cells and can produce cytokines such as IFN-γ in the presence of additional stimuli such as IL-18 provided by virus-infected cells (e.g., macrophages); NK cell activities prompt the lysis of viral-infected cells and the activation of cell-mediated immune responses. Type I IFNs can be produced by, virtually, all virus-infected cells. However, we have shown that reovirus-infected mast cells induce a more robust and heterogeneous IFN response compared to epithelial cells. DCs represent an important source of IFNs, however, they are not considered a longer-term local source of these cytokines because of their migration to secondary lymphoid organs, following infection, for antigen presentation. Therefore, long-term, tissue-resident mast cells are likely to be an important and sustained local source of IFNs below epithelial surfaces along with tissue-resident macrophages. For the purpose of clarity, the role of effector cells such as T cells and NKT cells involved in antiviral immune responses was not included in this figure. ncNK: non-conventional NK cells; cNK: conventional NK cells; DC: dendritic cells.

Epithelia represent a physical barrier that prevents the entry of invading pathogens into the body. Interactions between epithelial cells and pathogens result in the production of multiple mediators, including IFNs and CXCL8, intended to prevent invasion and activate immune responses [35,36]. Multiple viruses have evolved mechanisms to disrupt the epithelial barriers, gaining acces to tissues containing sentinel innate immune cells, such as macrophages and mast cells, which attempt to prevent the spread of pathogens. The role of mast cells in host antiviral responses is not well defined. Recently, our group has shown that cord blood-derived human mast cells activated with either reovirus or poly(I:C), a dsRNA mimic, selectively recruit human NK cells within a population of peripheral blood mononuclear cells via a CXCL8-dependent mechanism [28]. The production of this chemokine by mast cells was induced even in response to low doses of reovirus. However, a similar chemokine response was not observed in skin or airway fibroblasts, suggesting that mast cells may be particularly important in responding to early viral infection, through promoting NK cell recruitment. In addition, mast cell mediators produced in response to reovirus, specifically type I IFNs, induced NK cell activation, determined by enhanced CD69 expression and IFN-γ production in the presence of IL-18. Functional evaluation of NK cells exposed to mediators released from virus-infected mast cells showed that they also had increased cytotoxic activity against the target cell line K562 *in vitro* [33]. Using a subcutaneous matrigel model in SCID mice, we found that reovirus-infected human mast cells induced both the recruitment and activation of mouse NK cell to sites of infection [33]. Other viruses recognized by mast cells may have similar impacts as shown by St John *et al*., who demonstrated NK cell recruitment in response to dengue virus-infected mast cells *in vivo* [27], albeit at very high viral doses. Additionally, murine mast cells activated with poly(I:C) or CpG ODN, a pathogen-associated DNA, have been described to upregulate IFN-γ production by NK cells in a cell contact-dependent manner involving OX40L expression on mast cells *in vitro* [37]. Together, these data suggest that mast cells serve as sentinel cells in tissues exposed to the external environment and contribute to host antiviral responses, at least indirectly, by recruiting resting NK cells to sites of infection. Once NK cells have been recruited, their cytokine production and cytotoxic activities can be upregulated by mediators released from virus-infected or viral product-activated mast cells (Figure 1). It remains unclear whether mast cells will modulate the activities of the recently described tissue-resident NK cells found in the skin, uterus, intestine, and lungs [38,39,40]. The impact of mast cells on the development of “memory NK cells” also needs further clarification.

### 2.2. Cancer

Elevated numbers of mast cells can be observed either at the peri-tumoral or intra-tumoral level where they have frequently been described to be pro-tumorigenic via enhancing tumor angiogenesis. However, in some cases, the presence of mast cells has been associated with favorable tumor characteristics and good prognosis (Table 1). 

These contrasting mast cell contributions on tumor growth may result from the specific cytokine environment and the hypoxic-derived metabolites observed in a significant number of tumors. The balance of proteases observed in local mast cell subpopulations may also be a factor. There are different subtypes of mast cells containing tryptase or both tryptase and chymase in addition to carboxipeptidase A and cathepsin G-like protease. These might drive differing roles for mast cells in cancer as some of these proteases have been associated with tumor progression and angiogenesis [60,61,62]. Some authors have demonstrated that targeting of mast cell-derived mediators or mast cell receptors involved in pro-tumorigenic activities resulted in tumor development inhibition [63,64,65,66], suggesting that mast cells may be a potential therapeutic target in cancer. On the other hand, the recognized role of mast cells in mobilizing dendritic cells to draining lymph nodes [19,21], in recruiting and activating NK [27,28,33] and CD8^+^ T cells [22,67], as well as inhibiting the suppressive activity of Treg cells [68] suggest that triggering tumor-associated mast cell immune activities might be convenient as a potential immunotherapy for cancer.

In contrast, the role of NK cells in both tumor surveillance and tumor regression is better established. For example, individuals with defective NK cell function have a higher incidence of several types of cancers [69,70,71,72,73]. The anticancer functions of NK cells include acting as a source of IFN-γ, which activates many aspects of immune responses such as promoting the activities of cytotoxic T lymphocytes, NK cells, DC, and macrophages. It can also upregulate the presentation of tumor-associated antigens by inducing the expression of MHC class I molecules [74]. Importantly, it can block the differentiation and function of immunosuppressive cells [75,76] and regulatory cytokines [77,78]. IFN-γ production is crucial for preventing metastasis and is reported to inhibit tumor cell growth by multiple direct and indirect mechanisms [79,80]. This function may be particularly important during tumor surveillance. Despite the reported positive actions of IFN-γ, a further understanding of its activities and signaling is required as this cytokine has also been reported to favor cancer progression [81]. Another NK cell key function is the perforin and granzyme B-dependent cellular cytotoxicity of tumor or virus-infected target cells. Therefore, NK cells are potentially a powerful tool for use in immunotherapeutic approaches. However, NK cell activities can be negatively impacted, and their effective function restricted in the tumor microenvironment. Tumor cells escape effective immune surveillance by mechanisms involving the downregulation or upregulation of activating or inhibitory NK cell ligands [82,83] and the establishment of a tolerogenic microenvironment mediated by local hypoxia and by recruiting immunosuppressive cells, such as myeloid-derived suppressor cells, tumor-associated macrophages, and Treg cells, into the tumor microenvironment. These activities limit the cytotoxic roles of effector immune cells such as NK cells and CD8^+^ T cells [84]. Additionally, NK cells have been reported to poorly migrate to some tumor sites [43,85] which are not necessarily actively producing relevant chemokines and may be poorly vascularised. The importance of NK cell mobilization is reflected by several studies showing that the number of tumor-infiltrating NK cells correlates with the outcome of a variety of malignant tumors [86,87,88]. Therefore, current immunotherapies for cancer treatment involving NK cells are focused on restoring NK cell activities and on increasing NK cell infiltration into tumors.

**Figure 2:**
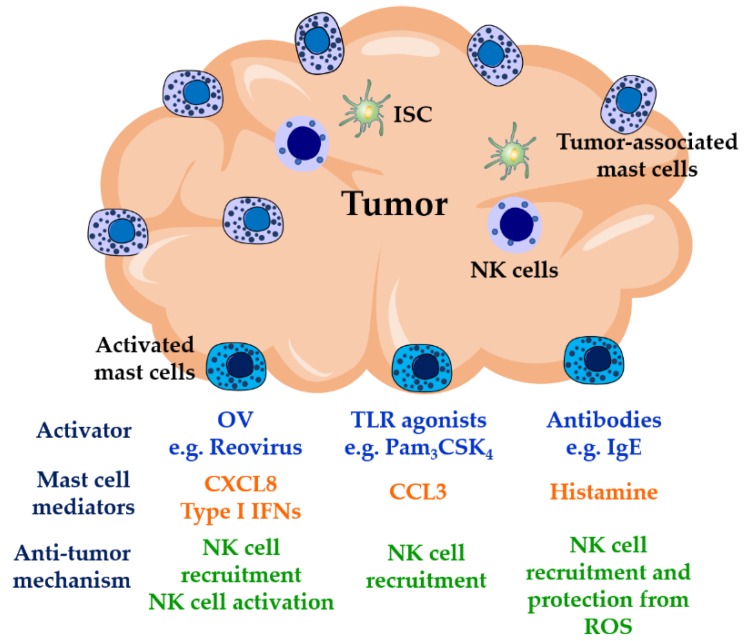
Mast cell-mediated NK cell anti-tumor activities. NK cells are poorly recruited to solid tumors where their activities are usually impaired by the presence of ROS released by myeloid cells and by the activities of immunosuppressive cells such as T regulatory cells, myeloid-derived suppressor cells, and tumor-associated macrophages in the tumor environment. Mast cells are found at high densities in solid tumors, where they show pro- or anti-tumorigenic activities. Mast cell location on tumors along with their modulatory activities on NK cells, therefore, may represent an important target in immunotherapies for cancer. Triggering mast cell function at tumor sites by OVs, TLR agonists, or IgE antibodies could result in proper recruitment and activation of NK cells. OVs: oncolytic viruses; TLR: Toll-like receptors; ISC: immunosuppressive cells; ROS: reactive oxygen species.

NK cell immunotherapies include adoptive cellular immunotherapy (ACT) and oncolytic viruses (OVs) associated with NK cells [89,90,91,92]. ACT involves the isolation and *ex vivo* activation of autologous or haploidentical NK cells which are then infused into patients to induce tumor regression [93,94,95,96]. Although ACT has shown successful results in patients with hematologic malignancies [97,98], poor results have been observed in the targeting of solid tumors, mainly because of the poor trafficking and infiltration of NK cells into the tumor. In contrast, OVs can penetrate, replicate inside the tumor, and kill tumor cells while leaving healthy cells relatively unharmed [99,100,101]. One of the OVs which has been examined in cancer immunotherapies is reovirus type 3 Dearing, which has been tested in clinical trials in several countries [102,103,104,105]. This is just one of several oncolytic therapies being tested or in clinical use. In addition to directly killing transformed cells, indirect anti-tumor actions of OVs rely on the recruitment and activation of effector immune cells. It has been shown that reovirus infection of tumor cells indirectly induces the recruitment and activation of NK cells via DC by mechanisms involving the production of cytokines such as IL-12 and type I IFNs *in vivo* and *in vitro* [89,106,107]. Gujar *et al*., showed that reovirus infection of prostate cancer cells induces the production of pro-inflammatory cytokines which could result in the trafficking of immune cells to the tumor site where reovirus is replicating [108]. Mast cells have the ability to initiate antiviral responses that include the promotion of NK cell recruitment and activation [28,33]. Therefore, we suggest that the use of oncolytic viruses that promote local mast cell production of type I IFNs would drive the upregulation of NK cell cytotoxic activities and IFN-γ production (Figure 2). Such mast cell-driven responses could potentially act in synergy with cytokines such as IL-12 derived from surrounding DCs [89]. It is important to note that host–OV interactions or the actions of NK cells recruited to tumor sites can be negatively modulated by tumor-derived immunosuppressor cytokines such as TGF-β, known to downregulate both mast cell and NK cell activities [109,110,111]. In the tumor, mast cells represent a source of this cytokine as well as of the proteases that can cleave and activate latent TGF-β [112,113]. Two independent groups have reported succesful effects on tumor regression when OV therapy is combined with inhibitors of TGF-β signaling [114,115], suggesting promising implications for OV-based immunotherapies. In addition to reovirus, OVs currently used in cancer therapy include herpes simplex virus [116], vesicular stomatitis virus [117], Newcastle disease virus [118], and vaccinia virus [119], all of which are known to activate mast cells [120,121,122,123]. Although viral infections are expected to induce IFN responses, it would be of interest to confirm the production of these cytokines, in addition to chemokines involved in the recruitment of effector cells with antitumor roles, by mast cells both *in vitro* and *in vivo.* Oncolytic virus therapies have been shown to improve tumor infiltration of transferred antigen-specific T cells, resulting in the eradication of established solid tumors [124]. Similar approaches could enhance the delivery of activated/modified NK cells during ACT as a result, in part, of mast cell activation. 

Pathogen or pathogen product activation of mast cells alone, locally within the tumor site, may also enhance NK cell function. Given the enrichment of mast cells at tumor sites and close association with blood vessels, local mast cell activation can be pivotal in reducing tumor growth and metastasis. For example, TLR2-mediated mast cell activation reduced tumor growth in several mouse models [66]. In mice in which the mast cells in the tumor microenvironment did not express TLR2, while other cells retained TLR2 expression, the ability of a TLR2 agonist to inhibit tumor growth was ablated [66]. A number of oncolytic strategies have included intra-tumoral injections, in some cases with marked abscopal impacts. The ability of mast cells to activate NK cells may be important in these processes and require further investigation. Since activated mast cells have also been demonstrated to enhance DC mobilization to draining lymph nodes [19,125], they may enhance both local innate NK cell functions and longer-term systemic-acquired immune responses to tumors simultaneously.

A major question for immunologists is whether allergic reactions serve an evolutionary purpose. Epidemiological studies have suggested a positive correlation between allergic diseases and a lower risk in the development of some tumors [126,127,128]. IgE antibodies have been reported to be the most abundant isotype in head and cancer tumor tissues [129]. Furthermore, elevated levels of tumor-specific serum IgE have been observed in patients with pancreatic cancer compared to control subjects [130]. IgE binds to Fc epsilon receptors expressed on a variety of immune cells; some of them are usually associated with tumors (e.g., eosinophils, basophils, NK cells, DCs, macrophages, and mast cells). These antibodies effectively induced antibody-dependent cellular cytotoxicity against the transformed cells [130,131]. Antigen-induced aggregation of IgE bound to FcεRI can result in the release of histamine through mast cell degranulation. IL-2-activated NK cells express the histamine receptor H4 which induces NK cell chemotaxis [132,133], that might be towards the tumor site if specific anti-tumor antibodies are present. In the tumor environment, the release of reactive oxygen species (ROS) by myeloid cells into the extracellular space downregulates T cell and NK cell activities followed by apoptosis [134]. These actions have been shown to be prevented by histamine via inhibition of NADPH oxidase, involved in the production of ROS [135,136]. Therefore, histamine in combination with IL-2, an NK cell-activating cytokine, has shown promising results in the activation of anti-tumor immune responses [137,138]. In a phase three clinical trial, Brune *et al*., described that immunotherapy with IL-2 supplemented with histamine dihydrochloride in patients with acute myeloid leukemia in complete remission improved leukemia-free survival compared to a control group [139]. The effects of histamine on both NK cell recruitment and activity-protection suggest that similar results might be observed in solid tumors. Therefore, the presence of mast cells in tumors provides a potential target in cancer immunotherapy. Activation of these cells with oncolytic viruses, TLR products, and/or through their FcεRI may result in the recruitment and activation of NK cells (Figure 2), mobilization of DCs to lymph nodes, and recruitment of T cell subsets [140]. In addition, they may enhance the immune response locally to OVs therapy.

### 2.3. Allergic Asthma

Allergic diseases such as atopic dermatitis and asthma are characterized by the development of Th2-associated cellular responses and the production of allergen-specific IgE antibodies [8]. Th2-derived cytokines such as IL-4, IL-5, and IL-13 contribute to the clinical manifestations of allergy. Although viral infections are associated with the development of Th1 responses, infection with respiratory syncytial virus (RSV) results in inflammatory responses mediated by Th2 cells that promote the production of Th2-derived cytokines and virus-specific IgE [141]. Severe RSV infections in young children that require hospitalization may confer a long-term risk for both asthma and allergic sensitization [142]. It has been suggested that RSV infections early in life may damage the growing lung, resulting in later bronchial obstructive symptoms. These responses may not be limited to RSV; rhinovirus has also been associated with asthma and wheezing in infants [143]. Whether there is a causal relationship between such viral infections and asthma development or whether infection and asthma have shared risk factors is not entirely clear. 

Mast cell activation, which may result from cross-linking of their IgE receptor [144], complement product mediated-activation [145] or as a result of local hypoxia [7] and elevated adenosine levels, has been associated with RSV infection. Tryptase, a marker of mast cell degranulation, was detected in bronchoalveolar lavage samples from infants hospitalized with RSV [146]. An additional marker of mast cell activation, the 9α,11β–prostaglandin F_2_, was present at higher levels in infants with RSV-induced bronchiolitis [147]. Al Afif *et al*., showed that human mast cells can become infected with RSV [148]. Although RSV-treated mast cells showed low levels of RSV antigen protein expression, these cells produce a variety of chemokines, including CXCL10 and CCL4, in a type I IFN-dependent manner [148]. These responses could result in the recruitment and activation of effector immune cells, such as NK cells, to the lungs. 

The role of NK cells in the development and effector phase of allergy remains controversial. Similar to CD4^+^ T cells, NK cells can polarize into different cytokine-producing subsets *in vitro*. In the presence of IL-12, NK cells produce IFN-γ (NK1 subpopulation), while IL-4-activated NK cells produce IL-4, IL-5, and IL-13 cytokines (NK2 subpopulation) [149]. IFN-γ production by NK cells plays an important role in antiviral immune responses [74]. Impaired production of this cytokine may result in failure to appropriately activate cellular-mediated immune responses and in the development of Th2 responses. Kaiko *et al*., demonstrated that NK cells and IFN-γ deficiencies during primary RSV infection *in vivo* lead to the development of viral-specific Th2 effector cells and subsequent allergic lung disease by a mechanism dependent on enhanced expression of IL-25 by epithelial cells [150]. These Th2 cells persisted in the long term as memory cells and could be reactivated by a secondary viral infection. These data suggest that impaired NK functions and/or NK cell recruitment to the lungs may be a predisposing factor in the development of viral-associated asthma. NK cells have also been shown to play a role in allergic responses beyond viral infections [151,152]. In a murine model of OVA-induced allergic airway inflammation (OVA-AAD), Mathias *et al*., reported that depletion of NK cells significantly impaired the development of OVA-AAD by preventing the secretion of Th2 cytokines, the generation of OVA-specific IgE antibodies, and the infiltration of T cells and eosinophils to the lungs [153], suggesting that NK cells play a pro-inflammatory role in allergic disease. 

**Figure 3:**
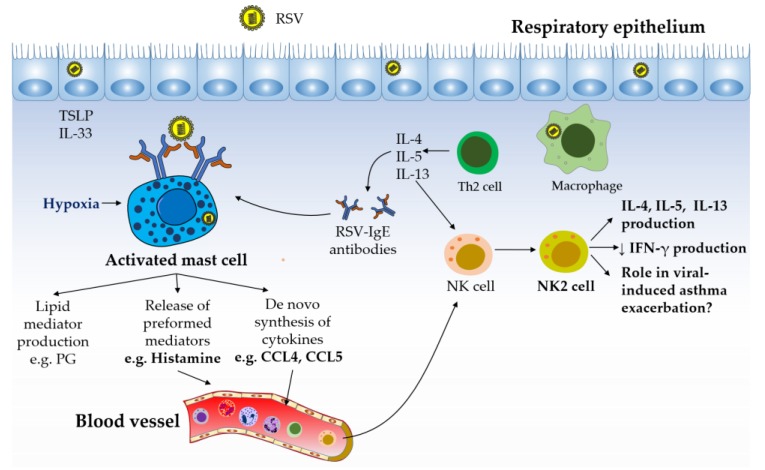
Mast cell–NK cell interactions in the pathology of allergic asthma. The production of Th2 cytokines and IgE antibodies has been reported to play a key role in orchestrating, perpetuating, and amplifying the inflammatory responses observed in allergic diseases such as asthma. RSV is described to evade host antiviral activities by eliciting Th2-skewed immune responses. Mast cell activation during RSV infection, possibly triggered by cross-linking of their IgE receptor by RSV, hypoxic conditions, and/or by direct infection with RSV, can lead to the production of the NK cell chemotactic factors CCL4 and histamine. Recruited NK cells are likely to differentiate into NK2 cells under the Th2 environment present in the lungs. NK2 cell-derived cytokines (e.g. IL-4 and IL-13) can contribute to the pathology of asthma by promoting Th2 responses and by downregulating IFN responses, possibly favoring virus-induced asthma exacerbations.

Once asthma has been stablished, the cytokine environment related to this pathology can favor the differentiation of Th2 cytokine-producing-NK cells. Wei et al., have reported high levels of IL-4-producing NK cells compared to IFN-γ-producing NK cells in asthmatic patients [154]. A similar trend has been observed in subjects with allergic rhinitis and atopic dermatitis [155,156]. However, it should be noted that elevated IFN-γ has also been reported in the BAL of asthmatic subjects [157]. The production of Th2 cytokines by NK2 cells might be associated primarily with asthma exacerbations, which are responsible for a significant proportion of the mortality associated with asthma [158]. Current asthma therapies are not fully effective in their prevention. Asthma exacerbations are commonly caused by respiratory viruses, such as RSV and rhinovirus [159], and are attributed to dysfunctional host antiviral responses [160], which have been associated with the prominent presence of asthma-associated Th2 cytokines [161]. IL-4 and IL-13 have been shown to impair the production of the antiviral cytokines type I and type III IFNs in bronchial human epithelial cells and plasmacytoid DCs by mechanisms involving the downregulating of viral sensors such as TLR3 and TLR9 [162,163,164]. Mast cells express receptors for Th2 cytokines. However, it is not yet known whether these cytokines can modify mast cell IFN responses. In addition to their antiviral activities, both type I and type III IFNs have been reported to efficiently block the development of Th2 responses as well as to inhibit cytokine production from fully differentiated Th2 cells *in vitro* [165,166]. Djukanovic et al. showed in a phase 2 clinical trial that inhaled administration of recombinant IFN-β effectively prevented virus-induced asthma exacerbations in difficult-to-treat asthmatics, suggesting that, at least in this group, impaired IFN production is associated with the development of asthma exacerbations in response to infection with respiratory viruses [167]. During RSV infection, mast cells might induce the recruitment of NK cells to the lungs through chemokine production (RSV-infected mast cells) and/or histamine release (IgE receptor cross-linking ). In the context of asthma associated with Th2 responses, it is possible that recruited NK cells will produce IL-4, IL-5, and IL-13 over IFN-γ, contributing therefore to both the pathology of asthma and impaired antiviral responses (Figure 3).

Additionally, it has also been reported that in some settings NK cells might play a regulatory role by killing activated T cells under appropriate conditions [168]. Therefore, it will be of interest to explore more in detail how mast cells can modulate the activities of NK cells during asthma responses and whether mast cells’ responses to virus contribute to asthma exacerbations.

## 3. Conclusions

Mast cells are strategically located at surfaces constantly exposed to the external environment, where they can interact with pathogens and coordinate innate immune responses that involve the mobilization of immune cells. Although mast cell contributions to host antiviral responses are not well defined, we have shown that virus-infected mast cells can specifically recruit and activate conventional NK cells involved in the lysis of infected cells and in the activation of cell-mediated immune responses through the production of IFN-γ. Further studies are required to determine the impact of virus-activated mast cells in the generation or activities of memory NK cells as well as of non-conventional NK cells. Because of the prominent presence of mast cells in solid tumors and the poor presence of NK cells at these sites, the manipulation of mast cells to coordinate the recruitment of NK cells represents a potential target in cancer immunotherapy. However, the complex role and interaction of mast cells with NK cells also need to be carefully considered in the context of allergic disease and specifically as potential contributors in the worsening of allergic disease in the context of viral infection.

## Figures and Tables

**Table 1:** Mast cells in tumors.

Tumor	Role	Biological Action or Clinical Observation
Pancreatic adenocarcinoma [41] * Prostate [42]	Pro-tumorigenic	Angiogenesis via VEGF production
Colorectal cancer [43] Lung adenocarcinoma [44] Melanoma [45] Gastric cancer [46]	Pro-tumorigenic	Angiogenesis
Renal carcinoma [47]	Pro-tumorigenic	Impair anti-tumoral responses via IL-10 and TGF-β production
Muscle invasive bladder cancer [48]	Pro-tumorigenic	Negative correlation between TIMC numbers and patient survival
Hepatocellular cancer [49] * Gastric cancer [50]	Pro-tumorigenic	IL-17 expression
* Hepatocarcinoma [51] * Colon cancer [52]	Pro-tumorigenic	Recruitment of MDSC Increase the suppressive role of MDSC via IFN-γ and NO production
* Skin carcinogenesis [53]	Anti-tumorigenic	Recruitment of effector immune cells to the tumor site
Breast cancer [54]	Anti-tumorigenic	Presence of mast cells is a good prognosis marker
Renal cell carcinoma [55]	Anti-tumorigenic	Positive correlation between TIMC numbers and patient survival
Esophageal squamous cell carcinoma [56]	Anti-tumorigenic	Negative correlation between IL-17^+^ mast cells and tumor invasion
Ovarian cancer [57]	Anti-tumorigenic	Mast cell infiltration in tumors with high vessel density was associated with improved survival
B cell lymphoma [58] Pleural mesothelioma [59]	Anti-tumorigenic	TIMC associated to favorable clinical outcome

* murine data. VEGF, vascular endothelial growth factor; TIMC, tumor infiltrating mast cells; MDSC, myeloid-derived suppressor cells; NO, nitric oxide.
